# Interventions for QoL and Other Outcomes Among Caregivers of Older Adults With Visual Impairments

**DOI:** 10.1093/geroni/igab046.654

**Published:** 2021-12-17

**Authors:** Afeez Hazzan, Pamela Haibach-Beach, Lauren Lieberman, Jamia Williams

**Affiliations:** 1 State University of New York at Brockport, Brockport, New York, United States; 2 SUNY Brockport, State University of New York, Brockport, New York, United States; 3 State University of New York, Brockport, State University of New York, Brockport, New York, United States

## Abstract

Unpaid family caregivers play a critical role in the care of older adults with visual impairments (VI). Caring for older adults with VI requires much time and energy, often resulting in psychological stress and reduced quality of life (QoL). However, there is a paucity of data on the impact of caregiving on QoL and related outcomes among these caregivers. The purpose of this study was to conduct a scoping review examining issues of QoL, health, stress, burden, and barriers among unpaid caregivers of older adults (i.e. aged 60 years or more) with VI. The study aimed to summarize interventions for addressing these issues. This study followed the Arksey and O’Malley (2005) five stage approach for scoping reviews. We performed a search of published peer-reviewed articles available in PubMed, CINAHL Complete, and PsycINFO to identify relevant studies. Two reviewers conducted the screening of titles, abstracts, and full-texts. Of the 452 records identified, 24 were eligible for full-text screening and five articles met the final inclusion criteria. The following four themes were identified: (1) prevalence of QoL-related barriers among unpaid caregivers of older adults with VI; (2) adverse events among unpaid caregivers of older adults with VI; (3) interventions for unpaid caregivers of older adults with VI; and (4) potential impacts of intervention on unpaid caregivers of older adults with VI. These findings reveal a lack of interventions for unpaid caregivers of older adults with VI, despite the prevalence of QoL-related barriers and adverse events. Research addressing these issues are urgently needed.

